# Filamin C Truncating Variant Causes Severe Conduction Defects and Mild Cardiomyopathy

**DOI:** 10.7759/cureus.74631

**Published:** 2024-11-27

**Authors:** Yusuke Ebana, Mariko Komine, Takuro Nishimura, Tetsuo Sasano, Masayuki Yoshida

**Affiliations:** 1 Department of Medical Genetics, Institute of Science Tokyo, Tokyo, JPN; 2 Department of Cardiovascular Medicine, Institute of Science Tokyo, Tokyo, JPN

**Keywords:** atrioventricular block, filamin c, hypertrophic cardiomyopathy (hcm), pacemaker implantation, truncating mutations

## Abstract

Filamin C (FLNC), recently identified as a causative gene of cardiomyopathy, is widely expressed in cardiomyocytes and is involved in signal transduction between the sarcomere and the plasma membrane. In general, the FLNC truncating variant causes severe dilated cardiomyopathy. A 70-year-old female was referred to our hospital with advanced conduction defects and underwent pacemaker implantation. Cardiac MRI revealed mild hypertrophic cardiomyopathy. As her father also underwent pacemaker implantation due to a cardiac conduction defect, the presence of familial cardiac arrhythmia was suspected. A whole-exome sequencing identified the FLNC truncating variant (NM_001458.5 FLNC:c.592_593del, p.Cys198Argfs*40). We experienced an FLNC-related cardiomyopathy case with predominantly advanced conduction defects, which postulated that the variant mainly affected the conduction system.

## Introduction

Filamin C is a dimeric protein encoded by FLNC (7q32), which was originally reported as a causative gene of dominant myofibrillar and distal myopathy. Recently, FLNC has also been identified as a causative gene in the autosomal dominant form of various cardiomyopathies such as hypertrophic cardiomyopathy (HCM), dilated cardiomyopathy (DCM), restrictive cardiomyopathy, arrhythmogenic cardiomyopathy, and non-compaction left ventricle cardiomyopathy [1,2]. The prevalence of pathogenic variants of FLNC ranges from 1% to 8% in the cardiomyopathy subtype.

Filamin C is widely expressed in cardiomyocytes and is involved in signal transduction, connecting the sarcomere and the plasma membrane [3]. Its structure consists of an actin-binding domain at the N-terminus and a dimerization domain that forms a dimer at the C-terminus.

According to ClinVar, 378 pathogenic and 147 likely pathogenic variants have been identified. Among them, the variants contributing to phenotypes including cardiac disease are 328 pathogenic and 124 likely pathogenic ones. FLNC truncating variants (FLNCtv) were reported to be mainly identified in DCM, although missense or in-frame indel variants were found in other phenotypes [4]. So far, although two variants were reported to be causative for DCM and conduction defect, these cases were ones in which the main phenotype was DCM.

Here, we report our experience with an FLNC-related cardiomyopathy case who had predominantly advanced conduction defects as the main symptom.

## Case presentation

**Figure 1:**
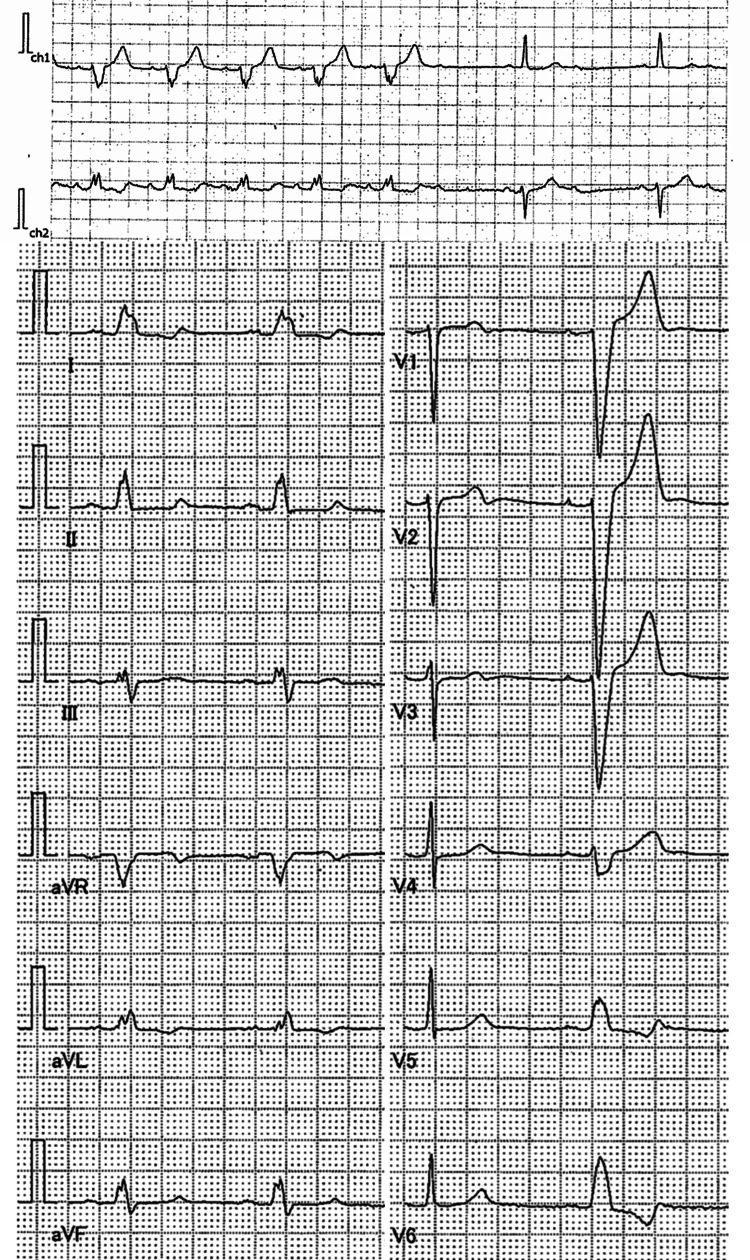
Electrocardiogram of the case The upper figure is a 2:1 atrioventricular block pointed out on our hospital admission. In the lower figure, a general ECG shows a complete left bundle branch block.

A 70-year-old female was referred to our hospital with an advanced heart block. For several years, she had often noticed palpitations and irregular heartbeats when she trained at the gym. When she visited a hospital, she occasionally felt lightheaded, and her physician noted bradycardia and diagnosed her with a 2:1 advanced atrioventricular block on an electrocardiogram. She was transferred to our hospital (Figure 1).

**Figure 2:**
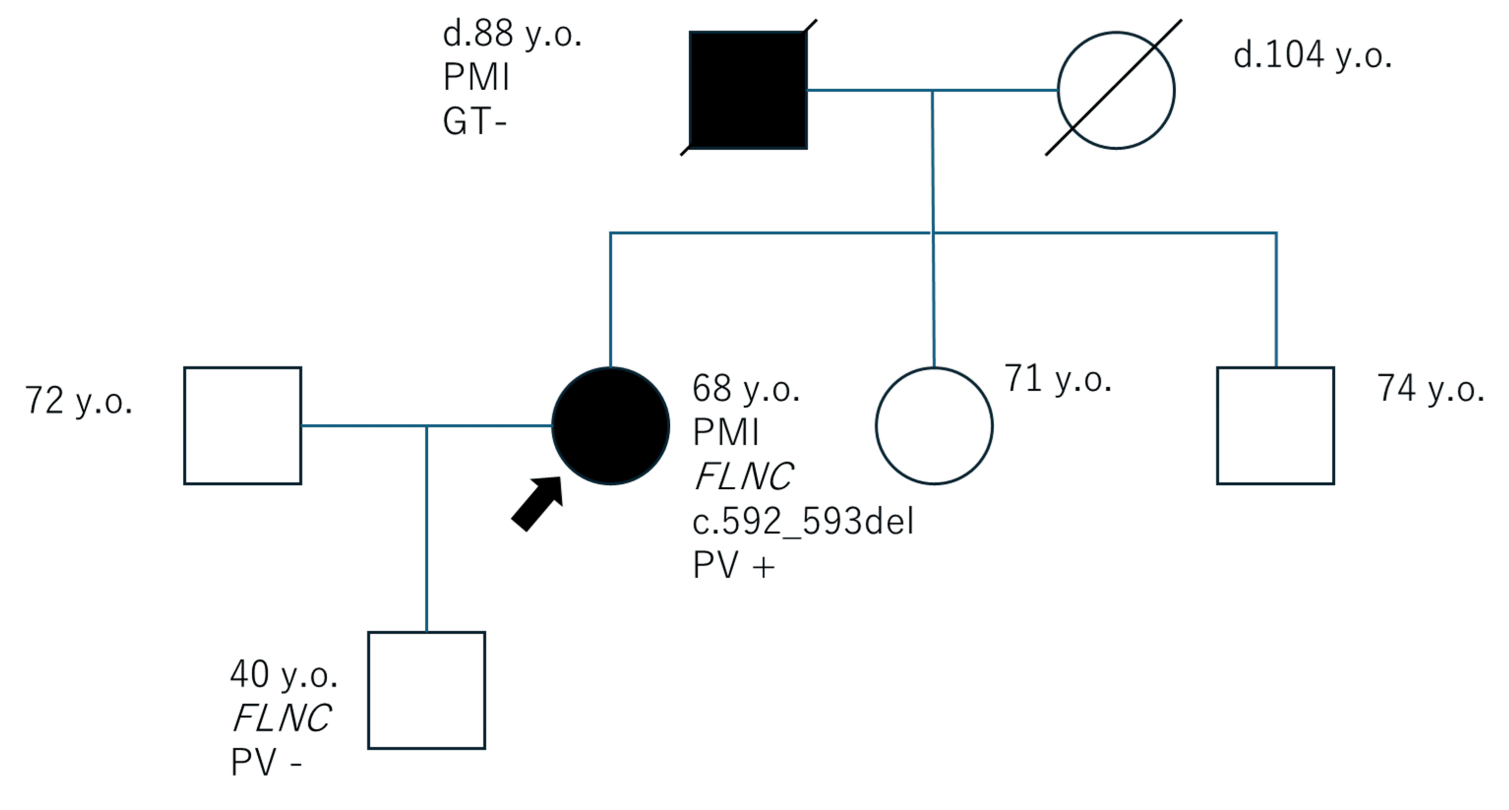
Family tree of the case The proband (arrow) and her father underwent pacemaker implantation. GT: genetic testing, PMI: pacemaker implantation, PV: pathogenic variant

The patient had a history of hypertension and food allergies and was currently undergoing treatment for these conditions but no myopathy. The patient's father had a pacemaker implanted, and her sister had a heart disease, but the details are unknown (Figure 2).

**Figure 3:**
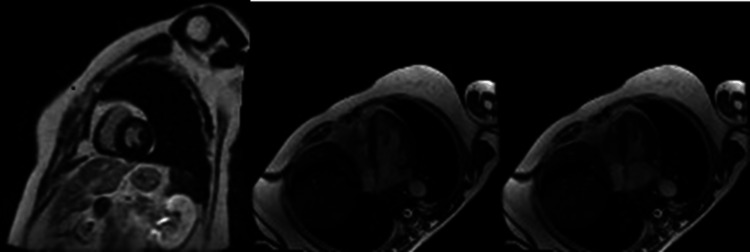
Cardiac MRI Cardiac MRI showed mild left ventricular hypertrophy. The right ventricle had no abnormality. MRI: magnetic resonance imaging

The electrocardiogram showed a 2:1 atrioventricular block and left bundle branch block. On 24-hour ECG monitoring, a 2:1 atrioventricular block was observed at bedtime. The basic rhythm was sinus rhythm, and the number of total heartbeats was 80,662 per day. Blocked atrial premature contraction followed by narrow QRS atrioventricular conduction was also observed. No lethal ventricular arrhythmia was detected. Laboratory data revealed mildly elevated B-type natriuretic peptide levels (24.2 pg/ml). The complete blood count, liver function, and kidney function were normal. The echocardiogram detected mild hypertrophy without dyssynchrony. Cardiac MRI showed mild concentricity and dilatation of the left ventricle, including a region of reduced T1 value in the ventricular septum. There were no findings suggestive of inflammation or edema. Late gadolinium enhancement was negative (Figure 3). She underwent a pacemaker (DDD mode) implantation.

As there was a possibility of secondary cardiomyopathy, genetic testing was performed. Genomic DNA is enriched for targeted regions using a hybridization-based protocol and sequenced using Illumina technology. All targeted regions are sequenced with > 50x depth. Reads are aligned to a reference sequence (GRCh37). The genetic analysis revealed associated variants in five genes (Table 1).

**Table 1:** Identified variants in the case

Gene name	Variant	Amino acid substitution	Interpretation
FLNC	c.592_593del	p.Cys198Argfs*40	Pathogenic
DMD	c.379A>G	p.Ile127Val	Uncertain significance
DMD	c.657T>A	plAsp219Glu	Uncertain significance
GATAD1	c.524A>G	p.Gln175Arg	Uncertain significance
SNTA1	c.1504G>A	p.Gly502Arg	Uncertain significance
GAA	c.1726G>A	p.Gly576Ser	Benign
GAA	c.2065G>A	p.Glu689Lys	Benign

Only FLNC (NM_001458.5):c.592_593del, p.Cys198Argfs*40 was a pathogenic variant. The deletion causes a stop codon to occur after the extension of 40 amino acids. This variant is located at the calponin homology domain 2 in FLNC, which results in the truncating structure that does not contain Rod1, Rod2, or the dimerization domain. The remaining ones were variants of uncertain significance or benign ones. This case was diagnosed as FLNC-related cardiomyopathy. The genetic testing of her healthy son was negative.

## Discussion

FLNC, the filamin C coding gene, is one of the causative genes involved in HCM but is most prominent in DCM. This gene is highly intolerant to loss-of-function (LOEUF: 0.25). FLNCtv was reported to be identified in DCM, although missense or in-frame indel variants were found in other phenotypes [4]. The phenotype of FLNC-related cardiomyopathy is a late-onset type, typically occurring in the 40s [5].

However, the case had a truncating variant, but the phenotype was mild HCM and advanced conduction impairment. The cases with FLNCtv who had cardiomyopathy with atrioventricular block were identified in an Icelandic population-based analysis [6]. The identified variant is located in an actin-binding domain. A case with a different variant in the same domain had DCM and a left bundle branch block [7]. It was also reported that left ventricular hypertrophy or dysfunction is not necessarily obvious in FLNCtv cases [8].

Filamin C, whose expression is enriched in cardiomyocytes and even more so in skeletal muscle, is responsible for mechanical transduction between cells by adhesion of the Z-disc of the sarcomere to the sarcolemma and the interventricular plate [9]. On the intervening disc, filamin C is located at the fascia adherens, where the ends of the myofibers reach the sarcolemma, close to the position of the desmosomal junction. Fragile filamin C may cause dysfunction of intercellular junctions. Therefore, FLNCtv could cause conduction disturbances. It is also postulated to regulate cytoskeletal protein organizations in response to stress. Aggregation of filamin C in the cytoplasm has been shown to play a pathogenic role [3]. This feature could cause a late onset of FLNCtv.

## Conclusions

We experienced an FLNC-related cardiomyopathy case that had predominantly advanced conduction defects. In general, cases with FLNCtv show a severe DCM or HCM phenotype, and this case was considered rare. More data regarding FLNCtv is required.
